# Menstrual hormone-induced cyclic thumb CMC instability and degeneration in women: a systematic review

**DOI:** 10.1186/s13293-022-00438-y

**Published:** 2022-06-20

**Authors:** Emily A. Parker, Alex M. Meyer, Ignacio Garcia Fleury, Joseph A. Buckwalter

**Affiliations:** Department of Orthopedics and Rehabilitation, University of Iowa Hospitals and Clinics, 200 Hawkins Drive, Iowa, IA 52242 USA

**Keywords:** Musculoskeletal sex differences, Thumb osteoarthritis, Trapeziometacarpal degeneration, Trapeziometacarpal instability, Thumb CMC arthroplasty

## Abstract

**Background:**

Relaxin is a hormone which peaks during the luteal phase of the menstrual cycle, and a known collagenolytic promoter that has been shown to avidly bind tissues supporting the trapeziometacarpal (TMC) joint in women. We hypothesize a causal linkage between cyclic binding of relaxin to the supporting tissues of the female TMC joint; and to the earlier onset of more severe TMC osteoarthritis (OA) commonly seen in women.

**Methods:**

A systematic literature review was performed per PRISMA guidelines, qualitatively and quantitatively assessing papers regarding relaxin–TMC joint stability interactions. The primary outcome variable was TMC joint degeneration/loss of function; the “late stage” consequences of relaxin-induced instability. The secondary outcome variable was presence of early signs of relaxin-induced instability; specifically asymptomatic TMC joint laxity in young women.

**Results:**

In healthy young women, menstrual cycle relaxin peaks corresponded with asymptomatic TMC joint instability. Immunohistochemical studies of TMC arthroplasty patients showed avidly increased relaxin binding to supporting tissues around the TMC joint in women but not men. Demographic analysis of patients from the TMC arthroplasty studies show a predominantly female cohort, who were on average significantly younger than the male surgical patients.

**Conclusions:**

Each relaxin peak during the menstrual cycle can target receptors on the soft tissues supporting the TMC joint, including—critically—the main stabilizing ligament: the anterior oblique. The cyclic instability is typically asymptomatic for years after menarche, but causes cumulative chondral microtrauma. This likely causes the early-onset, high severity TMC joint OA clinically pervasive among female patients at orthopedic hand clinics. Further research is indicated to develop risk assessment strategies and potential interventional options before and after the onset of hormonal laxity-induced OA.

## Highlights

- It is widely recognized among hand surgeons that female patients present at a younger age for basal thumb osteoarthritis, with more severe degeneration.
- The little-known menstrual hormone relaxin, which peaks every ovulatory cycle, may be responsible for the female predominance of this pathology.
- The soft tissue support structures for the thumb are known to express relaxin receptors which, when activated, trigger lysis of collagen and other extracellular matrix components.
- Laxity in soft tissues supporting the thumb, such as the deep anterior oblique ligament, would result in joint instability and microtrauma.
- If women cyclically experience thumb instability and cumulative cartilage microtrauma from menarche through menopause, the female propensity to develop TMC arthritis is aptly justified.

## Introduction

**Fig. 1:**
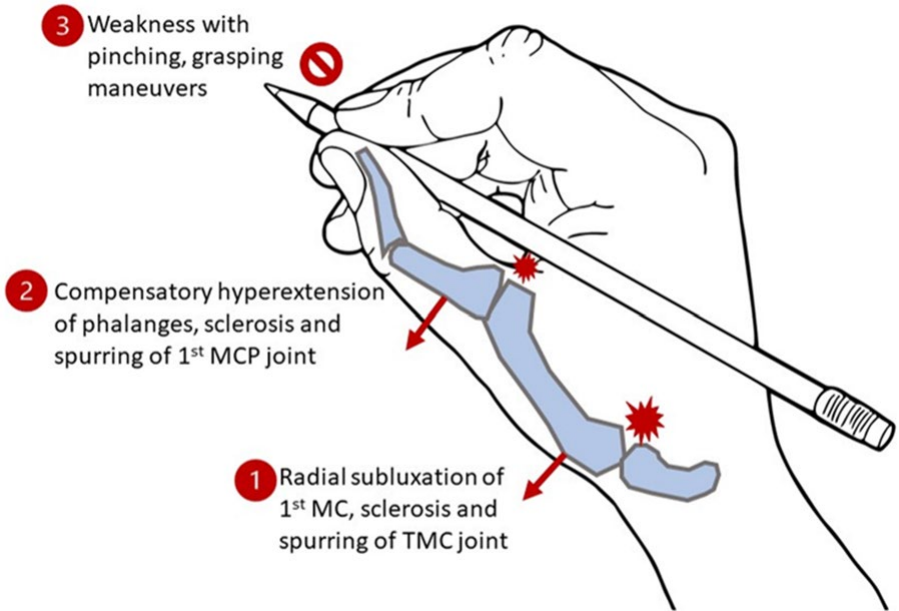
Legend, consequential pathology from long-term and/or repetitive TMC instability: The first site of pathology (1), the trapeziometacarpal (TMC) joint, will eventually sublux in the radial direction, causing chondral cartilage damage and creating a second site of pathology at the thumb metacarpophalangeal (MCP) joint. As the first phalanx is hyperextended in an attempt to compensate for the metacarpal subluxation relative to the carpal bone, the cartilage of the MCP joint becomes damaged (2). As a result, patients begin to notice marked weakness in the thumb, particularly with pinching and grasping tasks (3)

The unparalleled functional capacity of the thumb is facilitated by the unique structure of trapeziometacarpal (TMC) joint; a structure which increases the risk of OA development [1]. The TMC saddle joint has minimal intrinsic osseous stability, instead relying on attached ligaments for support, particularly the deep anterior oblique (palmar beak) ligament [1, 2]. The extensive number of relaxin binding sites around the female TMC joint incrementally decrease stability during child-bearing years [3–5].; post-menopausal women comprise 80% of the TMC joint OA patient population (Fig. 1) [6].

**Fig. 2:**
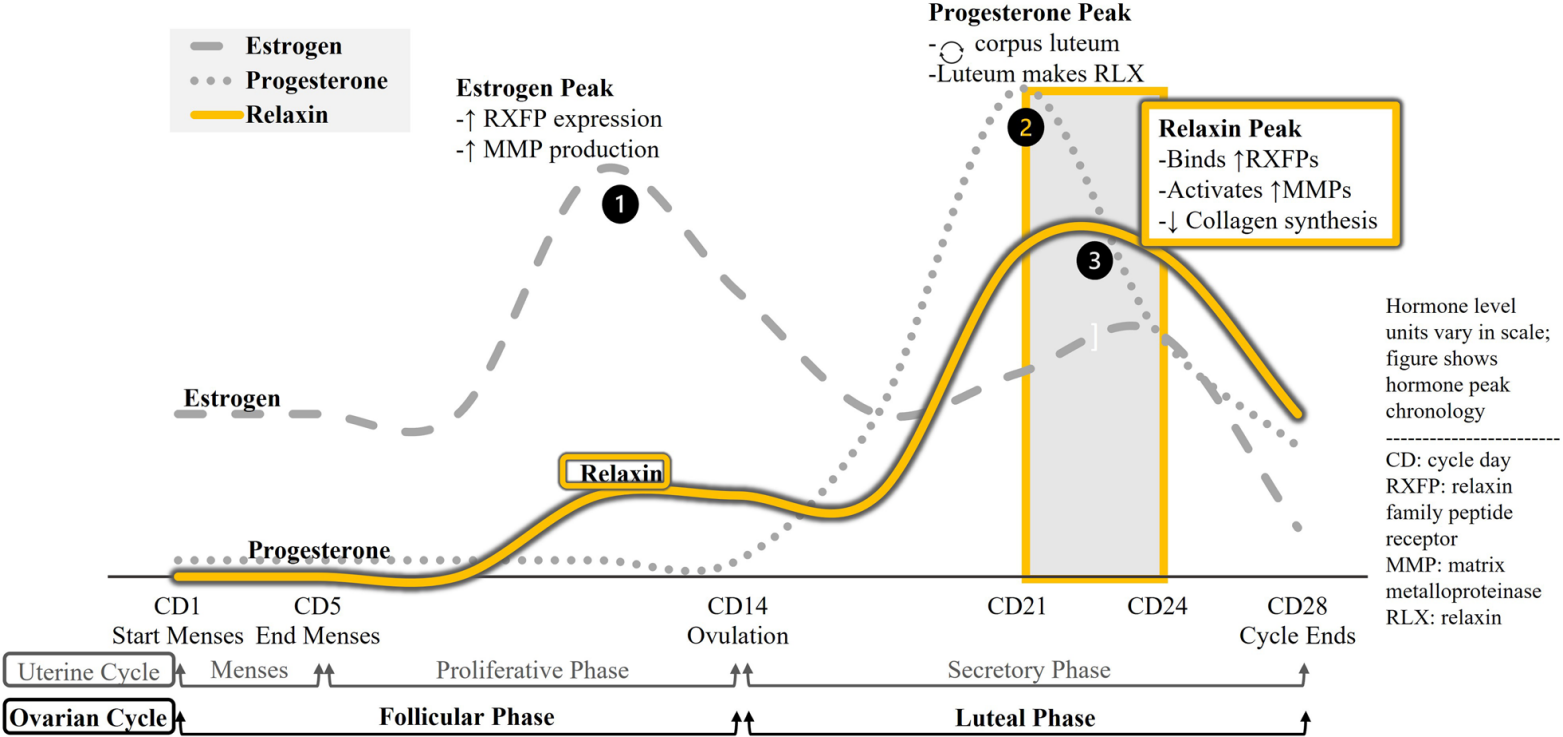
Legend, menstrual cycle hormone peaks and molecular effects: The sequence of hormone peaks for ovulatory menstrual cycles. Estrogen levels peak first, increasing expression of relaxin receptors in the body and increasing global synthesis of MMPs. The drop in estrogen triggers ovulation, and the remains of that ovarian follicle form the corpus luteum. As a temporary endocrine body, the corpus luteum secretes progesterone to prepare the endometrium for pregnancy and to sustain itself. It also synthesizes and releases relaxin, which binds receptors to activate MMPs recently upregulated by estrogen while also suppressing transcription of de novo collagen. Relaxin is active during the luteal phase, chiefly CD21-24

**Fig. 3:**
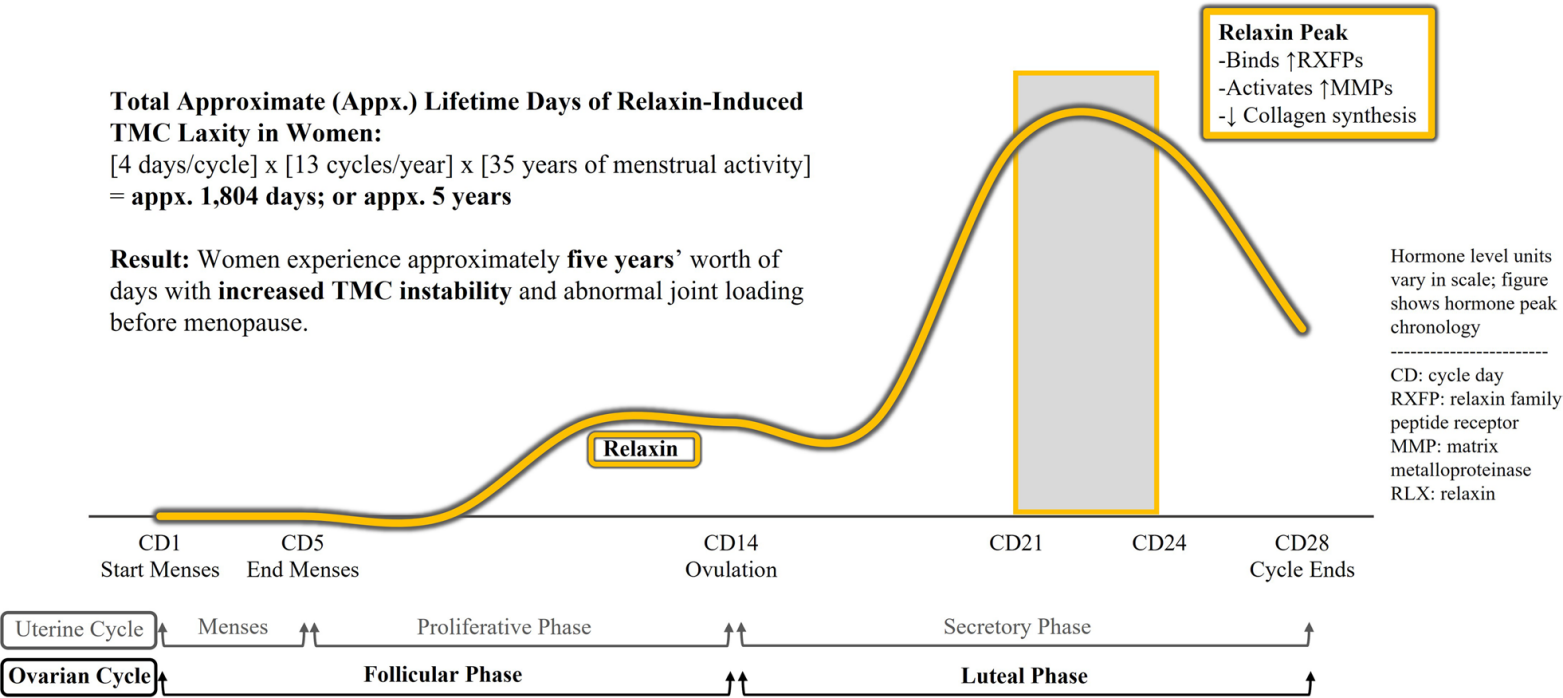
Legend, approximated cumulative lifetime instability: On average in the US, women reach menarche (start having ovulatory menstrual cycles) at age 14, and enter menopause (cease having ovulatory menstrual cycles) at age 49. Individual ages can vary widely. The luteal phase when relaxin peaks, CD21 to CD24, is consistent among women. With an average of 13 menstrual cycles per year and 4 days of instability per cycle, over 35 years of menstrual activity, women are subject to roughly 5 years of TMC joint instability before reaching age 50

Female reproductive hormone relaxin, noted for facilitating parturition, is infrequently discussed outside of obstetrics. In non-pregnant women, relaxin peaks during the luteal phase of the menstrual cycle, and it is critical to note that in women the hormone triggers collagen degradation (Fig. 2). The heavily reinforced pubic symphysis exemplifies this; in non-pregnant women, approximately 2600 pounds of force are required to separate the joint [7]. During pregnancy, relaxin facilitates joint separation via extensive collagen degradation [7]. Accordingly, it is concerning that women have receptors for this powerful hormone beyond the reproductive tract (Fig. 3) [3, 5, 8].

**Fig. 4:**
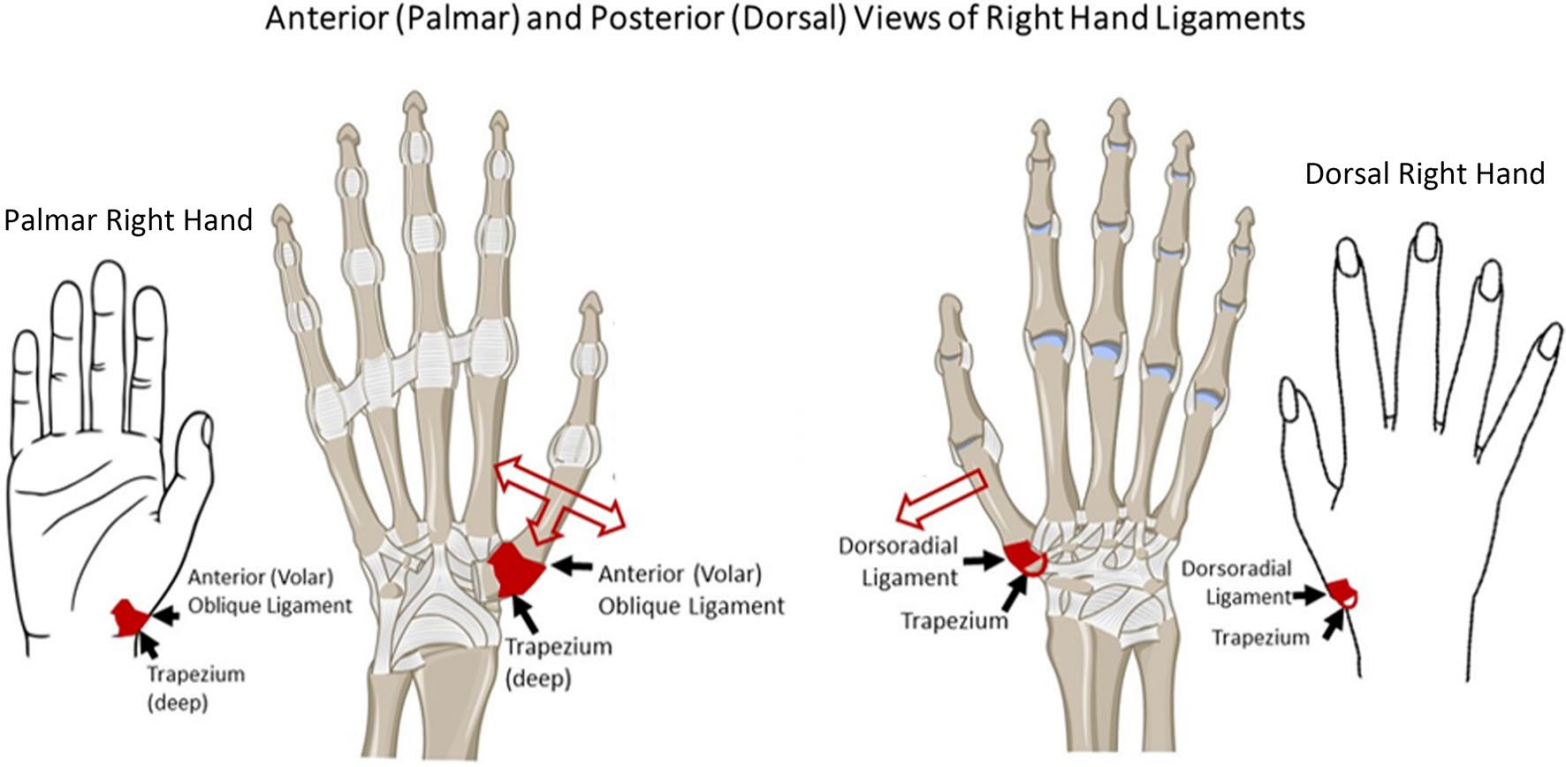
Legend, structual factors of the TMC joint detrimentally impacted by relaxin: The structures which compose the TMC joint or provide supplemental stability which contain relaxin receptors and may be degenerated in settings of elevated relaxin. Right: Dorsal view of the right hand. Relaxin attenuates the dorsoradial ligament, allowing lateral translation of the first metacarpal relative to the trapezium. Left: palmar view of the right hand. Relaxin attenuates the volar oblique ligament critical for TMC joint stability, allowing abnormal medial, lateral, and anterior (palmar) translation of the first metacarpal relative to the trapezium. Both: Trapezium chondrocytes contain relaxin receptors, resulting in direct cartilage damage in addition to damage caused by abnormal movement patterns

Known female-specific locations of relaxin family peptide receptors (RXFPs)—the anterior cruciate ligament (ACL), TMC joint tissues—portend common female pathophysiology, such as ACL tears and basilar thumb OA (Fig. 4) [3–5]. Despite TMC joint osteoarthritis (OA) being degenerative and ACL ruptures being acute, both show the harms of relaxin activity in periarticular tissues [3–5]. TMC joint OA can severely impact completion of activities of daily living, and quality of life. The present review aims to assess literature on relaxin and TMC joint OA, to evaluate female OA risk and prevalence, elucidate the hormonal aspects of OA development, and identify any potential preventive (pre-osteoarthritic) or interventional (after the onset of arthritis) options for management.

## Methods

### Literature Search

This literature search was refined from a prior Joanna Briggs Institute (JBI) protocol-compliant scoping review search for a separate manuscript, with broad parameters including any English-language literature discussing both relaxin and any musculoskeletal pathology and/or injury. It was noted during this earlier review that a significant number of results addressed relaxin and the trapeziometacarpal joint in women, leading to the present, focused systematic review.

The initial scoping review search strategies were developed by the authors and an orthopedic health sciences librarian with expertise in literature reviews in June 2020, with a repeat search for new literature relevant to the present review performed by the authors in December 2021. The initial search strategy was developed for MEDLINE. The first search string cast a wide net for any musculoskeletal injuries/pathology, with MeSH terms such as “hip injury” and “musculoskeletal injury”. Accompanying text words and phrases included “ligament” and “cartilage”. The second search string focused on female variations in relaxin hormone levels and activity, with MeSH terms such as “relaxin” and “relaxin receptor”. Accompanying text words and phrases included “cyclic hormonal variation” and “eumenorrheic”. The full search strategy for MEDLINE was as follows:

((((((((((((((((((hip[MeSH Terms])) OR (hip joint[MeSH Terms])) OR (hip injury[MeSH Terms])) OR (hip dysplasia[MeSH Terms])) OR (femoroacetabular impingement[MeSH Terms])) OR (non-arthritic hip pain[Text Word])) OR (hip dysplasia[Text Word])) OR (femoroacetabular impingement[Text Word])) OR (cam lesion[Text Word])) OR (pincer lesion[Text Word])) OR (acetabular labral tear[Text Word])) OR (musculoskeletal injury[MeSH Terms])) OR (musculoskeletal injury[Text Word])) OR (ligament[Text Word])) OR (ligament[MeSH Terms])) OR (cartilage[MeSH Terms])) OR (cartilage[Text Word]))

AND

(((((((((((relaxin[MeSH Terms]) OR (relaxin receptor[MeSH Terms])) OR (N(alpha) formyltyrosyl relaxin[MeSH Terms])) OR (hormone releasing intrauterine device[MeSH Terms])) OR (contraceptive agents[MeSH Terms])) OR (cycle, menstrual[MeSH Terms])) OR (dysmenorrhea[MeSH Terms])) OR (oligomenorrhea[MeSH Terms])) OR (amenorrhea[MeSH Terms])) OR (cyclic hormonal variation[Text Word])) OR (hormonal variation[Text Word]))

The search strategy was then adapted for EMBASE and CINAHL. Full search strategies for these databases are available upon request. The reference list of all included sources of evidence was screened for additional source materials via SCOPUS. All identified citations were uploaded to EndNote (EndNote X9.2, Clarivate Analytics, PA, USA) and duplicates removed by a combination of software screening and manual review. Titles and abstracts were then screened by two authors independently against the inclusion criteria, with animal-only studies being excluded. A full-text assessment was then performed to identify final inclusions.

Our initial scoping review screen yielded 82 included studies [8]. During the process of data extraction and analysis for the hip injury/relaxin scoping review, the authors noted a number of quality research studies addressing relaxin and TMC joint laxity/osteoarthritis in women. This information was condensed and presented in the scoping review as supporting evidence, and thus was not explored as a topic of focus. The authors decided to perform a more stringent systematic review screen of the 82 scoping review papers, selecting for papers specifically addressing the relaxin–TMC relationship, and excluding any lower quality literature, such as case studies. This “re-screen” was again performed independently by two junior authors, with oversight from the senior authors, in accordance with PRISMA guidelines.

### Statistical Analyses

Excel v.1808 (Microsoft Inc, Redmond, WA) was utilized to perform basic demographic calculations and all Student’s *t* tests. Student’s *t* tests were used to evaluate demographic data among all patients, specifically mean percent of female patients and mean patient age. They were also utilized for assessment of study outcome variables if data was present in sufficient amounts for an appropriately powered analysis.

### Outcome Variables

This group of studies facilitated a multi-outcome assessment of the impact of elevated relaxin on the female TMC joint. Our primary outcome was TMC joint degeneration and loss of function, evaluated via patients being indicated for/undergoing arthroplasty and/or radiographic evidence of osteoarthritis; and functional assessments, such as patient-reported outcome scores (PROs) or findings on physical exam in the clinic. Needing arthroplasty and/or severe degeneration on imaging was considered the “late stage” of detrimental relaxin effects.

Our secondary outcome, early symptoms of relaxin effects, evaluated the extent and impact of relaxin-induced TMC joint laxity in younger women and/or women with no significant osteoarthritis of the joint. The extent of relaxin-induced laxity had to be measured by an objective observer in a clinical/research setting, looking at passive and active range of motion, ability to resist applied stress, etc. The impact of the relaxin-induced laxity could be determined via PROs, subject failure during research tests of strength/stability, or changes in physical task metrics during periods of peak vs. trough relaxin levels (i.e., significant changes in grip strength).

Other outcome variables assessed are important considerations in current relaxin research: further elucidating the extent to which serum relaxin concentration (SRC) reflects ongoing relaxin activity, given its preference for paracrine signaling; and the extent to which the physiological location and concentration of relaxin receptors indicates a location of relaxin activity, particularly when comparing males vs. females. Evaluating the first point, paracrine vs. endocrine activity of relaxin at various serum levels, was best assessed in women with documented detectable SRC measurements, known relaxin receptors around the TMC joint, and documentation of TMC joint health. The second point, evaluating the location of relaxin receptors versus locations of known relaxin activity in women and men, was well-suited for examination in the present review Unlike the ACL, it is known that men have relaxin receptors present on TMC-adjacent tissue, yet do not experience early and/or severe osteoarthritis at rates similar to women.

Currently, the impact of relaxin has been well-documented from micro- to macro-level studies of ACL tears. The spectrum of evidence from molecular ACL degradation due to relaxin-activated MMPs, to population-level correlation of high relaxin levels and ACL tears, has constructed a sturdy base for future ACL/relaxin research. Therefore, with this review we aimed to determine what evidence is present or absent for TMC degeneration vs. relaxin levels; to see if pathways from relaxin peaks to injury or pathological state could be firmly established. We also assessed any proposed relaxin-focused interventions aiming to prevent injury/joint degeneration.

### Study Quality

Study quality was evaluated by two authors independently, prior to reaching consensus scores on the Modified Coleman Criteria Scale (MCMS). Oversight was provided by the senior authors. As a group, the six included studies had an average MCMS of 21.6 ± 12.2 (range: 28 to 58); ranking poor quality per MCMS categories. However, two of the immunohistochemistry-focused studies were notable outliers [3, 4], with MCMS scores at least 14 points lower than the remaining four studies which contained clinical information. With the two outlying studies removed, the average MCMS was 50.3 ± 6.8 (range 44 to 58); ranking fair quality per MCMS categories.

## Results

Of the 82 studies which met the criteria for inclusion in the authors' prior scoping review [8], six studies met criteria for inclusion in the systematic review of relaxin vs. TMC joint pathology [3, 4, 9–12]. All six studies assessed the location/properties of relaxin receptors around the TMC joint [4, 11, 12], and/or the pathophysiologic effects of relaxin on the TMC joint both early in life (joint instability) and later in life (joint osteoarthritis) [3, 9–12]. Except for the case–control study by Em et al. [9], all included studies were prospective cohort designs [3, 4, 10–12]. Three studies focused on TMC arthroplasty patients [3, 4, 11], two focused on benign joint hypermobility syndrome (BJHS) [9, 12], while the final study assessed hormonal contraceptive (HCP) use (Table 1) [10].

**Table 1:** Properties of Included Studies

Author, year	Title	Study type	Subject number (%F)	Mean age (years)	Subject factor(s) of interest	Interventions and/or information collected	Outcome variable(s)
Clifton et al. 2014	Detection of relaxin receptor in the dorsoradial ligament, synovium, and articular cartilage of the trapeziometacarpal joint	Prospective Cohort	15 (67%)	F 59 (44–79), M 67 (56–79)	Undergoing TMC arthroplasty	Collection of intraoperative tissue samples	Immunostaining for relaxin receptor in dorsoradial ligaments and synovium
Em et al. 2015	Serum relaxin levels in benign hypermobility syndrome	Prospective Case Control	88 (100%); 48 BJHS and 40 control	BJHS 25.2 + 6.6, control 25.4 + 7.1	Women diagnosed with benign joint hypermobility syndrome by the research team	Assessment for hypermobility (via Beighton, Brighton), serum relaxin levels	Average luteal SRC in women with BJHS vs. controls (all female); the presence of specific musculoskeletal pathologies in each group
Lubahn et al. 2006	Immunohistochemical detection of relaxin binding to the volar oblique ligament	Prospective cohort study	8 (100%)	Specified only as peri- or premenopausal	Non-menopausal women undergoing TMC arthroplasty, reconstruction of FCR	Collection of intraoperative tissue samples	Presence of relaxin receptors in volar oblique ligament
Pokorny et al. 2000	Self-reported oral contraceptive use and peripheral joint laxity	Prospective Cohort	55 (100%); 25 control and 30 test	Range 20–25	Women aged 20–25 on low-dose OCPs for 3 + months (test group) or not taking contraceptives for 3 + months (control group)	Passive anterior translation of tibia, 5th DIP hyperextension, and 2nd PIP abduction/adduction	Laxity of knee and hand between oral contraceptive users and non-users
Wolf et al. 2012	Serum relaxin is correlated with relaxin receptors and MMP-1 in the anterior oblique ligament	Prospective Cohort	49 (61%)	62 (43–78)	Patients undergoing TMC arthroplasty, trapeziectomy, and ligament reconstruction	Preoperative Beighton laxity score and serum relaxin level, intraoperative tissue sampling	Serum relaxin levels and relaxin receptor concentration in the anterior oblique ligament, relationship between MMP-1/MMP-13 and relaxin receptor location
Wolf, et al. 2013	Relationship of serum relaxin to generalized and trapezial–metacarpal Joint laxity	Prospective Cohort	289 (53%)	47 (18–91)	Healthy volunteers	Screening for hypermobility (Beighton), TMC stress X-rays, SRC testing	Serum relaxin levels and the effect on generalized and trapezial–metacarpal joint laxity

The 504 total included subjects were distributed unevenly among the studies, with a range of 8–289 and an average of 84 ± 105 subjects. The two largest studies, with 377 collective subjects, were assessing BJHS, while the three arthroplasty studies totaled 72 subjects (Table 1). In concordance with the preferential sex-specific manifestation of both BJHS and TMC OA, and as a result of artificial subject sex restriction by researchers, the average percent of female subjects per study was 80.2% ± 22.2%. Accordingly, as women tend to display symptoms of both conditions at a younger age, the average patient age was 44.0 ± 19.4 years (Table 1).

## Discussion

The present review aims to assess current literature regarding relaxin and TMC joint OA to better quantify the risk of pathophysiology among women, to determine a plausible mechanistic route for hormone related TMC OA development, and to pinpoint any promising strategies discussed for preventive or interventional purposes.

### Relaxin Overview

Analytical discussion of joint-specific relaxin effects requires adequate global knowledge of the hormone; its endocrine properties, receptor behavior, and general homeostatic/physiologic roles. Relaxin (RLX) is a peptide hormone present in both sexes, with preferentially paracrine activity. In women, the corpus luteum synthesizes most but not all relaxin, and prostatic tissue synthesizes relaxin in men [13–15]. Average serum relaxin concentration (SRC) between sexes is similar when women are not experiencing luteal phase peaks, but with relaxin often acting locally, SRC does not consistently reflect the extent of hormone activity (Appendix: Table 3) [15, 16].

Accordingly, the location of relaxin family peptide receptors (RXFPs) is a sensitive indicator of relaxin’s physiological roles. From a sex-specific musculoskeletal consideration, RXFPs are uniformly present on certain articular/peri-articular sites only in women, and RXFP expression is primed by estrogen and progesterone [14, 17–19]. Relaxin modulates ECM turnover, upregulating MMP-1/-13 (collagenases) and MMP-2/-9 (gelatinases) to degrade existing collagen while suppressing synthesis of new collagen via downregulation of transcription [14, 17–19]. Female relaxin levels peak in a temporal relationship with estrogen and progesterone menstrual cycle peaks (Fig. 2) [14, 20].

### TMC OA Risk, Prevalence, and Severity per Sex

The first aim of this review was to assess the prevalence and risk of TMC OA development in women vs. men. Two of the three TMC arthroplasty studies were mixed sex, not restricted by researchers (Clifton et al. [3]. and Wolf et al. [11]). Data from these 64 patients readily affirmed the sex-specific predominance of TMC OA, with the average percent of female vs. male patients being 64.0% ± 4.2% vs. 36.0% ± 4.2% (*p* < 0.05). Only Clifton et al. (*n* = 15) [3]. provided average age per sex, and despite the small number of patients, nearly reached significance when evaluating if women undergo surgical intervention at an earlier age compared to men (age 59.0 ± 7.8 years vs. 67.0 ± 5.8 years, *p* = 0.06).

Interestingly, all-subject analyses by Clifton et al. [3]. showed no correlation between extent of relaxin receptor staining and sex; and a lack of significant difference in receptor concentration between men and women. Similarly, the Eaton–Littler score of TMC OA severity was not significantly associated with sex. However, their analysis showed clinically relevant significance after first sex-stratifying patients, and then evaluating the male and female patient group Eaton scores vs. extent of relaxin receptor staining. While the male group did not show significant results, the female group had a strongly positive correlation between Eaton score and amount of relaxin receptors.

This supports prior hypotheses that men have protective factors (lower circulating SRC, androgen-associated cartilage protection) preventing laxity as the joint’s binding capacity for relaxin increases [3]. Patients with severe disease, requiring trapeziectomy, were all female in this study; significantly younger at time of surgery compared to the standard male patient, with the need for trapeziectomy having strong positive correlations with Eaton score and RXFP1 concentration (Table 2) [3]. Wolf et al. [11] did not sex-stratify their results, although patients were majority female (61%) (Table 1). In this study, increased SRC was significantly, positively correlated with expression of RXFP1 and MMP-1 (collagenase) in TMC joint peri-articular tissues (Table 2).

**Table 2:** Qualitative Review of Study Results

Author, Year	N (%F), Factor	Interventions; Outcome Variables	Hypotheses	Study results
Clifton et al. 2014	15 (67%) TMC arthroplasty patients	Tissue sampling Immunostaining for RXFP1 in dorsoradial ligaments and synovium	RXFP1 will be present in tissues stabilizing the TMC joint; therefore, relaxin could impact joint stability	Stratifying data by disease severity (Eaton–Littler score, timing to trapeziectomy) demonstrated a rapid pace of TMC degradation in women. Women were significantly younger at time of trapeziectomy, with a strong positive correlation of Eaton score and RXFP-1 concentration
Em et al. 2015	88 (100%) women; 48 with BJHS and 40 controls	Assessment for hypermobility, SRC Average SRC in women with BJHS vs. controls	Elevated relaxin may play a role in certain physiologic manifestations of BJHS	Serum relaxin levels of the BJHS group were non-significantly higher than controls (47.1 ± 20.3 pg/mL, 34.4 ± 22.1 pg/mL; *p* = 0.28). BJHS subjects had higher incidence of musculoskeletal pathologies, significantly (*p* < 0.05) arthralgia (33.3% vs. 25%), myalgia (55.6% vs. 27.5%), pes planus (57.8% vs. 30%), and hyperkyphosis (62.2% vs. 22.5%). The median relaxin levels were significantly greater in BJHS vs. control subjects with pes planus (33.3 vs. 16.4, *p* = 0.05) and/or hyperkyphosis (33.3 vs. 12.0, *p* < 0.05)
Lubahn et al. 2006	8 (100%) premenopausal women; TMC arthroplasty, FCR repair	Tissue Sampling Presence of relaxin receptors in volar oblique ligament	Female TMC OA prevalence may result from hormone-induced laxity of the volar oblique ligament. Relaxin is likely the hormone, it signals collagenases	Relaxin specifically bound all VOLs, with binding of cervical tissue > VOL > meniscal tissue. It was presumed that specific VOL binding of relaxin indicated cellular and/or extracellular matrix receptors. The lack of men presenting for treatment/study was noted
Pokorny et al. 2000	55 (100%) women aged 20–25; 3 months with 30 on low-dose OCPs (test), 25 not	AP tibial translation, 5th DIP extension, 2nd PIP abduction, adduction Knee and hand laxity in OCP users vs. non-users	The average joint laxity of women on OCPs will be higher than women not on OCPs, due to the endogenous estrogen and progesterone	Joint laxity had no significant difference between groups, including when stratified by cycle day groups. The control group had non-significant greater knee laxity during CD23+, and 2nd PIP laxity during CD12–22
Wolf et al. 2012	49 (61%) patients; TMC arthroplasty, trapeziectomy, ligament repair	Preoperative Beighton score and SRC, tissue sampling SRC, RXFP1 amount on anterior oblique ligament, relationship between MMP-1/MMP-13 and RXFP1 location	Relaxin is potentially involved in TMC joint laxity and eventual OA, via laxity of the anterior oblique ligament	Higher serum relaxin correlates with more thumb-area relaxin receptors and MMP-1 expression. Average mixed-sex expression levels were 3.73 pg/mL (0–9.45 pg/mL) for SRC, 5.23*10^6^ag for RXFP1 receptor, 0.022ag for MMP-1, and 0.318ag for MMP-3. Serum relaxin had significant relationships vs. log RXFP1 (*p* = 0.02); and vs. MMP-1 (*p* = 0.05). RXFP1, MMP-1, and MMP-3 were identified on the anterior oblique ligament
Wolf, J.M, Cameron, K.L. et al. 2013	289 (53%) healthy volunteers	Screening for hypermobility (Beighton), TMC stress X-rays, SRC testing SRC effect on general laxity, TMC joint laxity	High serum relaxin levels will correlate with laxity on TMC stress X-rays; it will also be associated with generalized joint laxity	42% of subjects had detectable SRC; 63% of women and 37% of men. SRC was 2.5 × greater in women, significant in 40–59yo’s. The all-ages female vs. male average SRC was 2.6 + 7.0 vs. 0.99 + 2.4, *p* < 0.05; with less significance when limited to detectable values. Women, particularly younger age groups, were more lax. SRC-detectable subjects had greater TM laxity, but only when controlling for age. Greater TM laxity was related to SRC, but so was younger age

### The Hormonal Aspect of Trapeziometacarpal Joint Osteoarthritis Development

The pathophysiology of osteoarthritis development can be a complex, multifactorial route. In the present review, the studies of younger, hypermobile women without TMC joint pathology were valuable for understanding early patterns of instability. Markers of early-onset TMC joint OA could potentially be discerned from such research, be it physical exam measures or laboratory/radiographic testing results [9, 12]. In addition, studies of women exposed to exogenous hormonal influences such as OCPs (Pokorny et al. [10]) allow hormone fluctuations to be viewed as a modifiable variable. Finally, clarifying the molecular-level mechanisms of OA development was facilitated by three studies analyzing tissues extracted during TMC arthroplasty (Table 2) [3, 4, 11].

In examining hormone-associated TMC joint OA development in women, it was found that the risk of CMC joint subluxation had a significant positive correlation with detectable SRC in young, healthy women [12]. High serum relaxin was also found to significantly correlate with increased expression of relaxin receptors and the powerful relaxin-activated collagenase, MMP-1, in the anterior oblique ligament of the CMC joint; one of the joint’s major structural stabilizers [11]. This correlation of serum relaxin, relaxin receptors, and MMP-1 concentration was detected in tissue samples from female TMC arthroplasty patients [11].

Therefore, a reasonable hypothetical pathway for the development of hormone-associated TMC joint OA in women begins with elevated serum relaxin levels. High SRC increases TMC joint subluxation risk at a young age, with subluxations having the potential to acutely damage chondral cartilage and worsen ligament laxity. As high SRC levels persist, the supporting tissues of the TMC joint express more receptors, binding more relaxin, which activates the increased number of local collagenases. Collagen degradation of the soft tissues supporting the TMC joint would further worsen joint laxity, allowing for increased subluxations and abnormal weight loading detrimental to chondral cartilage health (Fig. 1).

### Potential Preventive and/or Interventional Options to Mitigate Hormone-Related TMC Damage

Relaxin is necessary for correct function of the female reproductive tract, with undesirable extraneous effects beyond the reproductive organs, indicating that targeted, rather than systemic, treatments may be most promising [3]. It should be noted that hormonal contraceptives have been shown to reduce high relaxin levels and even decrease the rate of ACL tears among certain demographic groups, such as teen girls [21, 22]. However, only one study by Pokorny et al. [10] assessed impact of OCPs on laxity of the hand (second and fifth digits), and found no significant effects. Relaxin effects on women who are and are not using OCPs, in joints with well-established relaxin-mediated changes, such as the TMC joint, should be investigated.

The issue then becomes determining methods to locally decrease relaxin levels or counteract the pathophysiologic effects of relaxin peaks. Sustainability of the methods is a requirement, given that a woman reaching menarche and menopause at average ages, with average length menstrual cycles, will experience approximately 1800 days, or nearly 5 years, of lifetime luteal phase days during which her TMC joints could be at risk (Fig. 3).

While OCP use may not be desirable for some women, a low-cost but labor-intensive option for management is menstrual cycle tracking. The increased joint laxity occurs over a set span of days (cycle day 21 to cycle day 24) on average [23–25]. Therefore, higher risk days can be identified by tracking menstrual cycles via calendar or any number of free phone, computer, and tablet applications [24, 25]. This is likely most useful for women who are already showing signs of TMC pathology. They can consult with occupational therapists regarding activity restrictions, supportive bracing, or other practices to adopt during this phase of the menstrual cycle. Intervening at the point of mild pathology would ideally prevent or slow the progressive development of subsequent damage (Fig. 1) [11, 25].

As relaxin receptors have synovial localization, the TMC joint synovial tissue is a target for focused treatment in the future [26–29]. In contrast to the female reproductive hormones, androgens such as testosterone actually have a collagen-protective effect which almost directly opposes the mechanism of collagenolysis triggered by relaxin [30, 31]. As testosterone already has local application mechanisms (topical cream, implanted subdermal pellets), consideration of a directly-applied androgen treatment during peak relaxin days for women with moderate-to-severe TMC pathology is worth consideration.

### Perspectives and Significance

Common medical knowledge about relaxin is almost entirely limited to an obstetrical context, as is medical research about hormonal activity. It is critical to recognize that (1) Relaxin is physiologically active in women throughout a large span of their life, whether pregnant or not; and (2) Effects of relaxin are not limited to the reproductive organs in women; it is a powerful peptide hormone which modulates extracellular matrix turnover in numerous tissues throughout the body. A paradigm shift in medical care for women is necessary, to account for this significant physiological factor [8]. Early research should focus on discerning the tissues in which relaxin acts beyond the reproductive system; as the present review shows, a closer examination of female-predominant pathophysiology may yield answers here.

### Limitations

This was a systematic review of small sample size studies pertaining to the TMC joint and relaxin, increasing the risk of bias. Furthermore, the studies assessed both relaxin (serum concentration levels vs. immunohistochemical staining of local receptors), and TMC joint degradation (radiographic evidence of OA vs. surgical evidence of OA) via different measures, some likely to be more valid and reliable than the others.

## Conclusions

Each relaxin peak during the menstrual cycle can target receptors on the soft tissues supporting the TMC joint, including—critically—the main stabilizing ligament: the anterior oblique. The cyclic instability is typically asymptomatic for years after menarche, but causes cumulative chondral microtrauma. This likely causes the early-onset, high severity TMC joint OA clinically pervasive among female patients at orthopedic hand clinics. Further research is indicated to develop risk assessment strategies and potential interventional options before and after the onset of hormonal laxity-induced OA.

## Data Availability

The data sets used and/or analysed during the current study are available from the corresponding author on reasonable request.

## Appendix

See Table 3.

**Table 3:** Cellular/molecular effects of relaxin-relevant literature findings

Subcategory	Author, Year	Findings
Basic Properties of Relaxin	Goldsmith et al. 1995 [20] Grossman et al. 2010 [32] Lubahn et al. 2006 [4] MacLennan et al. 1983 [13] Powell et al. 2015 [14] Wolf et al. 2012 [11] Wolf et al. 2013 [15]	Relaxin (RLX^*^) is a peptide hormone in the insulin-like growth factor (IGF^†^) family Men and women have similar serum levels (400–500 pg/mL and 360–495 pg/mL), with luteal phase peaks in women Oral contraceptives decrease relaxin below a detectable serum level The major gene for relaxin in humans is H2; H2 relaxin binds relaxin family peptide receptor 1 and 2 The corpus luteum produces most relaxin, but synthesis occurs in the endometrium, placenta, breast tissue, and prostate Relaxin is biologically and immunologically active during pregnancy The capacity of relaxin to act locally means that serum levels do not always reflect activity
Properties of Relaxin Receptors	Bryant-Greenwood et al. 1982 [25] Dragoo et al. 2003 [11] Kapila et al. 1998 [18] Kleine et al. 2017 [33] MacLennan et al. 1983 [13] Powell et al. 2015 [14]	In humans, relaxin family peptide receptor-1 (RXFP^‡^) is most common, and has the highest affinity for H2 relaxin Relaxin binds receptors in a time-, temperature-, and pH-dependent manner RXFP^‡^ expression is primed by estrogen/progesterone in chondroblasts, fibrochondroblasts, myofibroblasts, and ligaments Estrogen-primed receptors can show maximum response at RLX^*^ levels 10–100 times lower than normal Estrogen, progesterone, and relaxin receptors modulate MMP^§^ transcription and post-translational modification Radioreceptor location detection is a sensitive indicator of the physiological roles of RLX^*^ Relaxin receptors are detectable in anterior cruciate ligament (ACL^¶^) remnants of female, but not male, surgical patients RLX^*^ binding was uniform, saturable, and specific to the synovial lining, stromal fibroblasts, and intima Relaxin receptors have been detected in the carpometacarpal joint of the thumb (1^st^ CMC^#^) in arthroplasty patients The synovial lining, dorsoradial ligament, volar oblique ligament, and articular cartilage cells had receptors Concentration of RLX^*^ receptors was significantly higher in women compared to men Relaxin receptors have been detected in the temporomandibular joint (TMJ^**^), on fibrochondrocytes and ligaments
Functional, Physiologic Properties of Relaxin	Ando et al. 1960 [34] Dragoo et al. 2003 [5] Galey et al. 2003 [35] Goldsmith et al. 1995 [20] Grossman et al. 2010 [32] Nose-Ogura et al. 2017 [21] Powell et al. 2015 [14]	Relaxin controls extracellular matrix (ECM^††^) turnover by stimulating collagen degradation, and suppressing synthesis Relaxin upregulates MMP^¥^ production, specifically collagenases (MMP-1/-13) and gelatinases (MMP^§^-2/-9) Active collagenases cleave tropocollagen, making it susceptible to subsequent denaturation by gelatinases The density and organization of collagen bundles, and total local collagen content decrease MMP^§^s induced by relaxin degrade collagen at a nanoscale level, and macro-level effects are not always appreciable Relaxin has dose-dependent and differential functioning; its effects depend on location and presence of other hormones There is a significant correlation between peak serum relaxin and peak serum progesterone levels Intracellular relaxin activates MAPK^‡‡^ and PI3K^§§^, increasing cAMP^¶¶^ and triggering vasodilation via MMP^§^-2/-9 Estrogen, progesterone, and relaxin binding synovial receptors upregulates inflammatory MMP^§^s, increasing OA^##^ risk Relaxin upregulates production of collagenases and gelatinases in ligaments and fibrocartilage During parturition, relaxin binding pubic ligaments dissociates collagen, increases water uptake, and decreases viscosity
